# Secreted protein acidic and rich in cysteine (SPARC) induces lipotoxicity in neuroblastoma by regulating transport of albumin complexed with fatty acids

**DOI:** 10.18632/oncotarget.12773

**Published:** 2016-10-20

**Authors:** Alexandre Chlenski, Marija Dobratic, Helen R. Salwen, Mark Applebaum, Lisa J. Guerrero, Ryan Miller, Gillian DeWane, Elena Solomaha, Jeremy D. Marks, Susan L. Cohn

**Affiliations:** ^1^ Department of Pediatrics, University of Chicago, Chicago, IL, USA; ^2^ Biological Sciences Division, Biophysics Core Facility, University of Chicago, Chicago, IL, USA

**Keywords:** neuroblastoma, SPARC, albumin, lipotoxicity, extracellular matrix

## Abstract

SPARC is a matrix protein that mediates interactions between cells and the microenvironment. In cancer, SPARC may either promote or inhibit tumor growth depending upon the tumor type. In neuroblastoma, SPARC is expressed in the stromal Schwannian cells and functions as a tumor suppressor. Here, we developed a novel *in vivo* model of stroma-rich neuroblastoma using non-tumorigenic SHEP cells with modulated levels of SPARC, mixed with tumorigenic KCNR cells. Tumors with stroma-derived SPARC displayed suppressed growth, inhibited angiogenesis and increased lipid accumulation. Based on the described chaperone function of SPARC, we hypothesized that SPARC binds albumin complexed with fatty acids and transports them to tumors. We show that SPARC binds albumin with Kd=18.9±2.3 uM, and enhances endothelial cell internalization and transendothelial transport of albumin *in vitro*. We also demonstrate that lipids induce toxicity in neuroblastoma cells and show that lipotoxicity is increased when cells are cultured in hypoxic conditions. Studies investigating the therapeutic potential of SPARC are warranted.

## INTRODUCTION

Neuroblastoma is notable for its broad spectrum of clinical behavior. Although excellent survival rates are observed in patients with low- or intermediate-risk disease, less than 50% of children with high-risk neuroblastoma are cured with current treatment approaches [1, 2]. Tumor histology is associated with survival, and favorable outcomes are observed in patients with neuroblastoma tumors that have abundant Schwannian stroma [3]. We previously reported that stroma-rich neuroblastoma tumors have decreased vascular density compared to stroma-poor tumors [4]. We have shown that Schwann cells produce Secreted Protein Acidic and Rich in Cysteine (SPARC), an extracellular matrix protein with anti-angiogenic activity [5]. We have also synthesized and tested SPARC peptides that structurally correspond to the follistatin domain of the protein and showed that they have potent anti-neuroblastoma activity [6, 7]. SPARC has also been shown to function as a tumor suppressor in other types of cancer, although its role in tumor pathogenesis appears to be context and cell-type dependent [8].

SPARC regulates the assembly, organization, and remodeling of the extracellular matrix (ECM), in part by functioning as an extracellular chaperone, binding components of the ECM and facilitating transport from the extracellular to the intracellular space [9]. In this study, we investigated the effects of stroma-derived SPARC on tumor growth using a novel neuroblastoma model that mimics stroma-rich neuroblastoma tumors. As expected, we found that tumors with high-levels of stroma-derived SPARC exhibited decreased tumor growth and inhibited angiogenesis. In addition, we detected a significant accumulation of lipids in the tumors. SPARC is known to influence fat metabolism in other systems. In *Drosophila*, inactivation of SPARC causes lethal malformation of the fat body [10] and SPARC knock-down mice have increased numbers of adipocytes and larger adipose tissue pads [11, 12]. Further, serum levels of SPARC correlate with Body Mass Index [13], insulin resistance, diabetic retinopathy, and diabetic nephropathy in both human patients and experimental animals [13–17].

Dietary lipids are stored in adipocytes in the form of triglycerides (TG). For consumption in other tissues, TG are converted to free fatty acids (FA) and transported in the intravascular space by albumin [18]. To be utilized for energy in target tissues, albumin-bound FA must cross the blood vessel wall by active transport through the endothelial monolayer [19–21], although few details of the mechanism are known. Because SPARC is known to bind albumin [22], we hypothesized that SPARC may enhance the internalization and transcytosis of FA in endothelial cells by acting as a chaperone for albumin. To test this hypothesis, we evaluated the interaction between SPARC and albumin and determined that the binding affinity is sufficient for the two proteins to interact under normal physiologic conditions. We also show that SPARC increases the internalization and transcytosis of albumin in endothelial cells, and that both proteins co-localize in the cytoplasmic vesicles. Further, we found that FA are toxic to neuroblastoma cells in hypoxic conditions, suggesting that SPARC may induce lipotoxicity in neuroblastoma tumors.

## RESULTS

### Experimental stroma-rich neuroblastoma model

**Figure 1:**
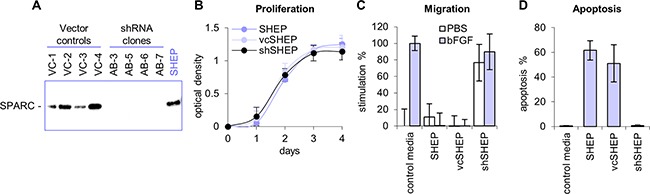
Inhibition of SPARC expression with shRNA **A.** SPARC shRNA vector was transfected into non-tumorigenic neuroblastoma SHEP cells, which express high levels of SPARC (shSHEP). Control SHEP cells were transfected with empty vector (control vcSHEP). SPARC expression was minimal to undetectable by Western blot analyses in the shSHEP clones. The level of SPARC expression in vcSHEP cells were similar to the parental SHEP cell line. **B.** shRNA inhibition of SPARC did not affect proliferation of SHEP cells. The proliferation rates of shSHEP clones, control vcSHEP, and parental SHEP cells were the same. **C.** Conditioned media from parental SHEP and vcSHEP cells potently inhibited endothelial cell migration towards bFGF *in vitro*. Conditioned media from the shSHEP cells lacked the ability to block migration towards bFGF and was stimulatory in the absence of activator. **D.** Conditioned media from the parental SHEP and vcSHEP cells strongly induced endothelial cell apoptosis *in vitro*. shRNA inhibition of SPARC expression in the shSHEP cells blocked the ability of conditioned media to induce endothelial cell death. Data are shown as average for four clones ±SD.

Substrate adherent, non-tumorigenic (S-type) neuroblastoma SHEP cells share many characteristics with Schwann cells, including tyrosinase activity specific for melanocytes [23] and expression of fibronectin, vimentin, collagen IV [24], and SPARC [5]. To confirm that the tumor suppressor function of SPARC was maintained in this neuroblastoma model, shRNA was used to decrease expression in SHEP cells (Figure 1). In cells transfected with the shRNA construct (shSHEP), SPARC expression was inhibited to levels undetectable by Western blot analysis. The expression of SPARC in vector-transfected control cells (vcSHEP) was similar to the level detected in the parental cell line (Figure 1A). No significant difference in the proliferation rates of shSHEP, vcSHEP, and the parental SHEP cells was observed (Figure 1B), and none were able to form colonies in soft agar (data not shown). Conditioned media collected from the parental SHEP and four vcSHEP clones contained high levels of SPARC protein and potently inhibited endothelial cell migration and induced endothelial cell apoptosis. In contrast, inhibition of endothelial cell migration and apoptosis were not observed in experiments performed with conditioned media collected from four shSHEP clones with undetectable levels of SPARC (Figure 1C–1D).

**Figure 2:**
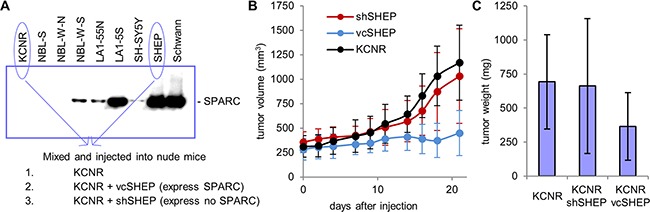
Experimental model of stroma-rich neuroblastoma **A.** Western blot shows that SPARC secretion is higher in less tumorigenic neuroblastoma cell lines. Conditioned media from Schwann cells is shown for comparison. Schwannian stroma-rich neuroblastoma tumors were modeled by injecting nude mice with a mixture of highly tumorigenic, highly angiogenic KCNR cells which express no SPARC. Stromal component was represented by SHEP cells with modulated SPARC expression, which are anti-angiogenic, non-tumorigenic and express high levels of SPARC. **B, C.** Three weeks following inoculation, large tumors developed in animals injected with KCNR alone or with mixture of KCNR and shSHEP cells, which express no SPARC. In contrast, tumors in mice injected with a mixture of KCNR and control vcSHEP cells, which express normal levels of SPARC, were significantly smaller.

To investigate the effects of stroma-derived SPARC on neuroblastoma tumorigenesis, we developed an *in vivo* model that mimicked Schwannian-rich neuroblastoma by mixing highly tumorigenic KCNR cells (neuroblastic or N-type cells) cells with non-tumorigenic SHEP cells. Neuroblastoma xenografts comprised of a range of KCNR to SHEP ratios were established, and we found that tumor growth was significantly inhibited with a 1:4 KCNR:SHEP ratio of cells (Supplementary Figure S1). In contrast, tumor growth was not suppressed in studies with KCNR:SHEP ratios of 1:1 or 1:2. We next established subcutaneous tumor xenografts using KCNR cells mixed with either shSHEP cells (SPARC-negative) or control vcSHEP cells (SPARC-positive) at a 1:4 ratio. Large tumors developed in animals injected with only KCNR cells (mean tumor size 693±329 g) or KCNR mixed with shSHEP cells with down-regulated SPARC (mean tumor size 662±470 g), whereas tumors in mice injected with KCNR mixed with vcSHEP cells were significantly smaller (mean tumor size 366±236 g, p=0.03) (Figure 2).

### Histologic characteristics of stroma-rich neuroblastoma model tumors

**Figure 3:**
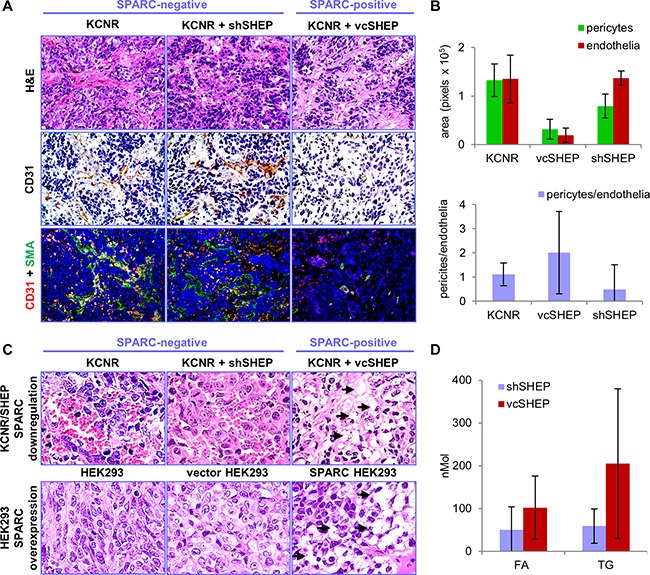
Histology of KCNR/SHEP tumors Inhibition of SPARC with shRNA suggests its role in angiogenesis, and lipid metabolism. **A.** Tumors from animals injected with KCNR alone or KCNR/shSHEP with down-regulated SPARC, were highly saturated with red blood cells. Control tumors from mice injected with the mixture of KCNR/vcSHEP, contained fewer red blood cells than KCNR alone or KCNR mixed with shSHEP tumors. Staining with CD31 shows increased angiogenesis and abnormal vessel morphology in tumors without SPARC expression. Magnification 400x. **B.** For quantification, endothelial cells were labeled with red fluorescent anti-CD31 antibody and pericytes were visualized with green anti-α-SMA antibody (also shown in panel A). The red blood vessel area was low in the tumors containing KCNR and vcSHEP cells. Anti-angiogenic properties of these cells were voided by shRNA inhibition of SPARC expression. In addition, pericyte coverage was increased in the presence of SPARC. **C.** Large number of droplets (shown by arrows) with deposited lipids was present in KCNR/vcSHEP tumors which express SPARC, compared to SPARC-negative KCNR and KCNR/shSHEP tumors (top panel). Similarly, increased deposition of lipids was apparent in HEK293 xenografts with overexpressed SPARC, compared to the SPARC-negative wild-type and empty vector-transfected HEK293 (lower panel), described in our previous studies [25, 40]. All panels show enlarged portion of H&E image at x400 magnification. **D.** Quantification of lipids in tumor tissues shows significant increase in free FA and TG in SPARC-expressing tumors (p<001).

Histological evaluation of the model stroma-rich neuroblastoma tumors demonstrated significant differences in angiogenesis (Figure 3A). SPARC-negative tumors were highly saturated with red blood cells contained in blood lakes and had scant stroma. In contrast, the tumor xenografts with high levels of stroma-derived SPARC contained fewer red blood cells and more stromal tissue. Image quantification showed that the blood vessel area was significantly decreased in tumors with high SPARC expression (Figure 3B). In contrast, the measured area of the blood vessels in the SPARC-negative tumor xenografts comprised of KCNR and shSHEP cells was similar to the level observed in xenografts established with KCNR cells alone. We also observed increased lipid deposition in the SPARC-positive tumors. Significant accumulation of lipids in the presence of SPARC was also present in other xenografted tumor models [25], while low amounts of lipids were detected in the SPARC-negative tumors (Figure 3C). Free FA and TG were quantitatively measured in five SPARC-positive and negative stroma-rich xenografts of similar size. Significantly higher levels of free FA and TG were detected in the SPARC-positive vs SPARC-negative tumors (2.03- and 3.46-fold increase, respectively, p<0.05) (Figure 3D).

### Interaction between SPARC and albumin

**Figure 4:**
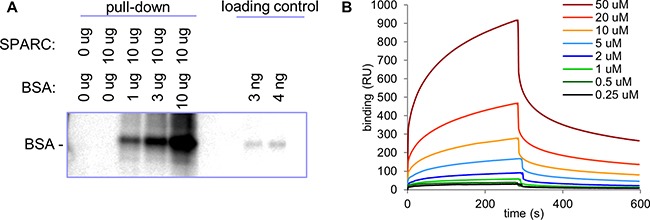
Analysis of SPARC/albumin interaction **A.** Indicated amounts of SPARC and albumin were immunoprecipitated with anti-SPARC antibody. SPARC-bound albumin was detected with anti-AF488 antibody. **B.** Histidine-tagged recombinant SPARC was used in SPR analysis as a ligand on an NTA sensor with indicated concentrations of BSA used as an analyte. Determined binding K_d_ was 18.9±2.3 uM.

To investigate if SPARC mediated the increased deposition of lipids in neuroblastoma tumors by binding and transporting albumin loaded with FA, we characterized the interaction between the two proteins. Using a pull-down assay we confirmed that SPARC and albumin interact in a concentration-dependent manner (Figure 4A). Surface Plasmon Resonance (SPR) analysis with histidine-tagged SPARC immobilized on a solid support demonstrated that SPARC binds albumin with a Kd=18.9±2.3 uM (Figure 4B). With the normal serum concentration of albumin in the range of 500-750 uM, this affinity is sufficient for a strong interaction between SPARC and albumin. Both human and bovine serum albumin (BSA) bound SPARC with similar affinity (data not shown), and all subsequent experiments were conducted with BSA.

### SPARC enhances internalization and transendothelial transport of albumin in endothelial cells

**Figure 5:**
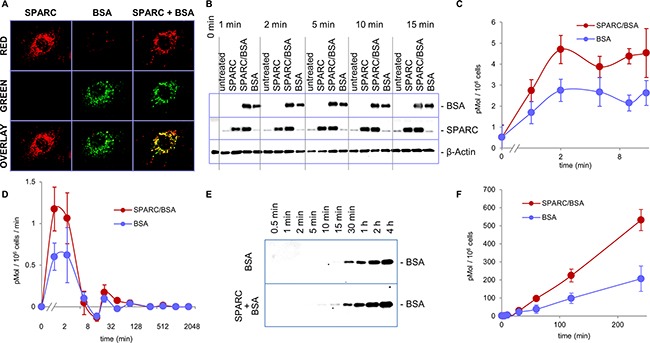
SPARC enhances internalization and transcytosis of albumin **A.** Red AF594 SPARC and green AF488 albumin were added to HUVEC cells, both proteins were internalized and co-localized inside the endothelial cells. **B.** HUVEC cells were treated with AF488 albumin alone or with SPARC as indicated. Internalization of exogenously added albumin was probed by Western blot with anti-AF488 antibody. Significant enhancement of albumin internalization was apparent within 1 minute of the treatment. **C.** Image quantification of albumin internalization. **D.** Combined data from several short-and long-term treatment experiments show that the rate of albumin internalization is increased by SPARC in the first 2 minutes. **E.** AF488 albumin added to the upper chambers of Transwell inserts with a HUVEC monolayer with and without SPARC. Transcytosed albumin was sampled from the bottom chamber at the indicated time points and detected by Western blot with AF488 antibody. **F.** Quantification of transcytosis by measuring the amounts of transcytosed albumin in the bottom chamber by Western blot.

To deliver FA from the intravascular space to target tissues, albumin is transported through the endothelial cell monolayer by transcytosis [20]. We previously demonstrated that SPARC functions as a chaperone for collagen I by directing its internalization into fibroblasts [9]. To investigate if SPARC serves the same function for albumin, we evaluated protein internalization in endothelial cells using green AF488 albumin and red AF594 SPARC. As shown in Figure 5A, significant co-localization of red and green fluorescence was seen in the intracellular vesicles in endothelial cells. Western blot analysis demonstrated that endothelial cells internalize exogenously added albumin in a time-dependent manner, with equilibrium observed in 10-30 min. (Figure 5B and 5C). In combination with SPARC, the rate of albumin internalization was significantly increased, especially in the first two minutes (Figure 5D). We further tested whether SPARC also increases transendothelial transport of albumin. Using a Transwell assay, we found that transport of albumin through the endothelial monolayer was time-dependent, and significantly increased in the presence of SPARC (Figure 5E and 5F). The high rate of albumin internalization in the first minutes of treatment may have especially significant impact in the dynamic conditions of blood flow, substantially increasing transendothelial transport.

### Lipotoxicity in neuroblastoma tumors

**Figure 6:**
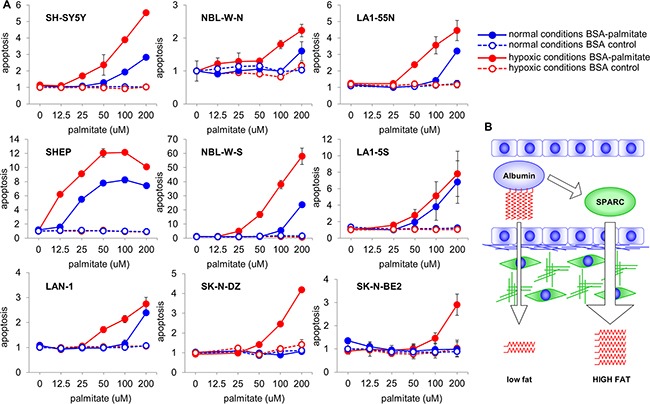
Lipotoxicity in neuroblastoma **A.** Effect of enhanced delivery of FA was tested in neuroblastoma cell lines treated with increasing amounts of albumin loaded with palmitate in normoxic and hypoxic conditions. While in normoxic conditions treatment with FA caused toxicity in some cell lines, in hypoxic conditions lipotoxicity was observed in all nine cell lines. For direct comparison, the data are shown as relative to the treatment with BSA control at 0 uM point. **B.** SPARC regulates lipid physiology and metabolism by enhancing delivery of FA to the target tissues. Without SPARC, lower amounts of FA-carrying albumin is transported to subluminal compartments. In the presence of SPARC, rate of albumin transcytosis is significantly higher, and larger amounts of FA are delivered to the target tissues.

It has been demonstrated that FA may be toxic to cancer cells [26], and accumulation of TG in lipid droplets is a protective mechanism against lipotoxicity [27]. We tested the toxicity of palmitic acid in nine neuroblastoma cell lines. Because β-oxidation of FA depends on an oxidative potential that may be limited in some regions of a tumor due to low levels of oxygen, the cell lines were treated in both normoxic and hypoxic conditions. As shown in Figure 6, in normoxic conditions palmitate caused moderate toxicity in some of the neuroblastoma cell lines. However, in hypoxic conditions, significant lipotoxicity was evident in all nine cell lines. For most cell lines the toxic concentrations of palmitate were in the range of 50 to 100 uM, which is significantly below the normal amounts of fatty acids in blood [28]. In contrast to neuroblastoma cells, hypoxia did not enhance lipotoxicity of palmitic acid in the normal cells tested under the same conditions (Supplementary Figure S2).

## DISCUSSION

In this study, we investigated the effects of stroma-derived SPARC on tumor growth using a novel *in vivo* stroma-rich neuroblastoma model. Consistent with previous studies, we found that stroma-derived SPARC suppressed angiogenesis and tumor growth. Interestingly, we also detected increased deposition of lipids in the tumors with stromal cells that express high levels of SPARC. To investigate if SPARC mediated the lipid deposition by chaperoning albumin-bound FA from the intravascular space to the tumor tissue, we tested the binding affinity of SPARC to albumin using pull-down assays and SPR analysis. We show that SPARC binds albumin at Kd=18.9±2.3 uM, and that SPARC increases the internalization and transcytosis of albumin in endothelial cells, supporting a regulatory role for SPARC in the delivery of albumin-bound FA to target tissues.

Recent studies show that FA may be toxic for many cancer cell types [26], and we found that palmitate induced lipotoxicity in neuroblastoma cell lines. We hypothesized that because catabolism of FA requires significant oxidative potential, lipotoxicity may be increased in the hypoxic conditions characteristic of *in vivo* solid tumors [29]. We found that palmitate induced significant lipotoxicity in neuroblastoma cell lines cultured in hypoxic conditions, suggesting that SPARC may promote lipotoxicity and neuroblastoma cell death by increasing the delivery of albumin-bound FA.

Lipid metabolism in cancer is strikingly different from normal cells. In most normal cells, FA are not catabolized or synthesized. Rather, dietary lipids are incorporated directly into cell membranes as needed for cell growth [30, 31]. In contrast, in cancer cells dietary lipids are actively catabolized to produce energy, and *de novo* synthesis of structural lipids is a hallmark of cancer metabolism. Although our studies show that lipotoxicity is induced by palmitate in normal cells cultured in normoxic conditions, hypoxia does not enhance this process. This type of lipotoxicity can be induced by endoplasmic reticulum stress caused by excessive incorporation of saturated FA into cell membranes [32]. In normal physiologic conditions, unsaturated FA protect the cells from this type of toxicity [29]. However, lipotoxicity is enhanced by hypoxia in cancer cells, indicating that mitochondrial stress through uncoupling of oxidative phosphorylation contributes to this process [33]. Further understanding of the difference in mechanisms of lipotoxicity in normal and cancer cells may ultimately lead to novel therapeutic targets. Indeed, FA have recently been shown to decrease the incidence and progression of neuroblastoma tumors in animal models [34, 35], supporting this hypothesis.

As the exclusive transporter of FA from adipose tissue to other tissues for consumption, albumin plays an essential role in lipid metabolism and physiology [18]. It is the most abundant plasma protein with a concentration in human blood of 42 g/l or 0.64 mM, which carries 0.7 molecules of FA on the average. Free FA are nearly insoluble in aqueous solutions, but when bound to albumin, FA exist in the vascular space at a high concentration of 0.4 mM [36]. Although FA represent the most abundant group of small hydrophobic molecules transported by albumin, many hormones and drugs are also transported and delivered to their intended sites by albumin [37]. Thus, SPARC may have additional therapeutic benefit by enhancing the delivery of other molecules transported by albumin to tumor tissues.

In conclusion, although the mechanisms by which SPARC regulates tumor growth are complex, we show the importance of the biological role of SPARC as a chaperone for serum albumin. By enhancing the transport of albumin from the vascular space to tumors, SPARC mediates lipid deposition in tumor tissue, leading to lipotoxicity. Further investigations of the therapeutic potential of SPARC for children with neuroblastoma are warranted.

## MATERIALS AND METHODS

### Cell culture and rhSPARC production

Neuroblastoma cell lines KCNR, NBL-S, NBL-W-N, NBL-W-S, LA1-55n, LA1-5s, LAN-1, SK-N-DZ, SK-N-BE2, SH-SY5Y, and SHEP were grown at 5% CO_2_ in RPMI 1640 (Life Technologies, Grand Island, NY) supplemented with 10% heat-inactivated FBS, 2 mM L-glutamine, and 1% penicillin/streptomycin. Primary Human Umbilical Vein Endothelial Cells (HUVEC) were purchased from Lonza (Walkersville, MD) and maintained at 37°C and 5% CO_2_ in EGM-2 media completed with SingleQuots/Bullet Kit (Lonza) and grown for no longer than six passages. Human embryonic kidney HEK293 cells (ATCC, Manassas, VA) transfected with SPARC were cultured at 5% CO_2_ in DMEM (Life Technologies) supplemented with 10% heat-inactivated FBS. For collection of the conditioned media, cells were washed three times with PBS and incubated with serum-free DMEM for 4 hours. The media was then replaced, conditioned for 72 hours and collected. Recombinant human SPARC (rhSPARC) was purified as described [25]. KCNR was a kind gift from Dr. Carol Thiele. LA155n, LA1-5s, SK-N-BE2, SHEP, and SH-SY5Y were kind gifts from Dr. June Biedler. LAN-1 was a kind gift from Dr. Robert Seeger. NBL-W-N, NBL-W-S, and NBL-S were established in our laboratory [38, 39]. SK-N-DZ was purchased from ATCC. All cell lines were authenticated by short tandem repeat (STR) profiling and profiles were found to be identical to known profiles for the cell lines. Authentication of KCNR, NBL-S, NBL-W-N, NBL-W-S, LA155n, LA1-5s, SHEP, and LAN-1 was performed at The Johns Hopkins University Fragment Analysis Facility (Baltimore, MD) using the AmpF*l*STR Identifiler PCR Amplification Kit (Applied Biosystems, Carlsbad, CA). SK-N-DZ and SK-N-BE2 were authenticated at ATCC using the PowerPlex 18D System (Promega, Madison, WI). All cell lines tested negative for mycoplasma contamination using the MycoAlert detection assay (Lonza). For proliferation assays, cells were plated in 96 well plates, grown for three days and assayed with the CellTiter 96AQ_ueous_ Nonradioactive Cell Assay Kit (Promega) according to the manufacturer's instructions.

### shRNA design and down-regulation of SPARC expression

The following shRNA oligonucleotides were designed to form a small hairpin complimentary to sequence GAACCACCACTGCAAACAC, located in the SPARC coding sequence 218 bp downstream of the translation start codon in the 6^th^ exon:

Upper: GATCCCGAACCACCACTGCAAACACTTCAAGAGAGTGTTTGCAGTGGTGGTTCTTTTTTCCAAA

Lower: AGCTTTTGGAAAAAAGAACCACCACTGCAAACACTCTCTTGAAGTGTTTGCAGTGGTGGTTCGG

After annealing, double-stranded insert was cloned into the BamHI/HindIII site of the pRNA-H1.1/Neo vector (GenScript, Piscataway, NJ). Recombinant plasmids were purified and integrity of the insert was verified by sequencing. The construct was transfected into neuroblastoma SHEP cells (shSHEP) using Lipofectamine 2000 (Life Technologies) according to the manufacturer's instructions; control SHEP cells were transfected with the same vector without the insert (vcSHEP). Individual clones were isolated by selection with hygromycin and the levels of SPARC expression in the media conditioned for at least 48 hours were analyzed by Western blot as described [5]. Proliferation and the effect of media conditioned by SHEP, and several shSHEP and vcSHEP clones on endothelial cell migration and apoptosis were analyzed as described previously [5].

### Western blot analysis

Lysates were prepared by boiling cell pellets in buffer containing 50 mM Tris-HCl pH 6.8, 2% SDS, and protease inhibitor cocktail (Sigma-Aldrich, St. Louis, MO) for 10 minutes. Conditioned media was supplemented with 0.5 mM PMSF, concentrated 50-100 times, and dialyzed against PBS using Centricon YM-3 filter units (Millipore, Billerica, MA) at 4°C. Protein concentrations were determined with the BCA Protein Assay Reagent (Pierce, Rockford, IL). Ten ug of total protein were electrophoresed on 4-20% SDS-PAGE gradient gels and transferred to nitrocellulose membranes. Membranes were blocked in TBS with 0.1% Tween-20 and 5% nonfat dry milk. Goat anti-SPARC antibody (R&D, Minneapolis, MN) was used at 1:1000 dilution and anti-AF488 antibody (Invitrogen, Carlsbad, CA) at 1:5000 dilution in blocking buffer. Blots were developed with anti-mouse-HRP secondary antibody and ECL Detection Reagent (Amersham, Piscataway, NJ).

### Xenograft studies

Schwannian stroma-rich neuroblastoma tumors were modeled by injecting female 4-6-week old homozygous athymic nude mice (Harlan, Indianapolis, IN) subcutaneously with a mixture of 2.5×10^6^ highly tumorigenic KCNR cells and 10^7^ of non-tumorigenic shSHEP cells transfected with SPARC or vcSHEP transfected with vector control. Tumor size was measured three times a week using a calipers, and tumors were harvested once KCNR controls were >1000 mm^3^. A portion was snap frozen and the remaining tissue prepared for immunohistochemical analysis. Xenografts were stained with hematoxylin & eosin (H&E) and for evaluation of angiogenesis with anti-CD31 antibody (Santa Cruz, Santa Cruz, CA) at a 1:100 dilution as described [6]. For quantitative analysis of angiogenesis 4-μm-thick sections were stained with anti-CD31 antibody at a 1:50 dilution followed by red fluorescent secondary antibody. To characterize the blood vessel architecture, pericytes were visualized with green fluorescence using anti-α-SMA antibody (Sigma-Aldrich) at 1:100 dilution. The area occupied by each cell type was quantified at x100 magnification in triplicate fields in each sample using ImagePro software (Media Cybernetics, Silver Spring, MD). Lipid accumulation was morphologically evaluated in H&E sections of KCNR/SHEP xenografts obtained in this study and in sections of HEK293 xenografts with modulated SPARC expression obtained in previous studies [25].

### Quantification of lipids

Levels of free FA in tumor tissues were determined with Free Fatty Acids Quantification Colorimetric/Fluorometric Kit (BioVision, Milpitas, CA) following the manufacturer's protocol. Briefly, 10 mg of snap frozen xenograft tumor tissue were homogenized using a Pellet Pestle Motor Tissue Grinder (Kimble-Kontes, Vineland, NJ) in 200 ul of 1% Triton X-100 in chloroform. The extracts were centrifuged at 17000 g for 10 minutes, organic phase was collected, air-dried at 50°C until evaporation of chloroform and then vacuum-dried for 30 minutes to remove residual chloroform. Dried lipids were dissolved in 200 ul of Fatty Acid Assay Buffer and 1 ul was used to determine the amounts of free FA by following the manufacturer's instructions. Levels of TG were determined using Triglyceride Quantification Colorimetric/Fluorometric Kit (BioVision) following the manufacturer's protocol. Briefly, 10 mg of snap frozen xenograft tumor tissue were homogenized in 100 ul of 5% NP-40 in H_2_O using a Pellet Pestle Motor Tissue Grinder, slowly heated to 100°C for 5 minutes and cooled down to room temperature. Heating/cooling was repeated one more time and samples were centrifuged at 17000 g for 2 minutes. The supernatant was diluted 10x with H_2_O and 1 ul was used to determine the amounts of TG following the manufacturer's instructions. Both free FA and TG were measured by colorimetric and fluorometric methods on an Infinite 200 Pro microplate reader (Tecan, Männedorf, Switzerland). Lipid amounts were determined by comparing with standards of known amounts of FA or TG. All measurements were done at least in triplicates and the data are shown as mean ± SE.

### Binding assays

To enable detection and to distinguish exogenous from endogenously produced albumin, a fluorescent tag was added to BSA (Sigma-Aldrich) using the Alexa Fluor 488 Microscale Labeling Kit (Invitrogen) following the manufacturer's protocol. For immunoprecipitation assays, 10 ug of rhSPARC were incubated with 0, 1, 3, and 10 ug of AF488-BSA at 4°C in 1xTBS and 1 mM CaCl_2_. After 2 hours, 10 ug/ml of goat anti-SPARC antibody was added for 2 hours followed by Protein A Agarose (Millipore) for an additional 2 hours. Complexes were eluted and Western blot was performed using rabbit anti-AF488 antibody for detection. The binding was evaluated in at least three different experiments. To determine affinity of the interaction, SPR was performed on a BIAcore 3000 (GE Healthcare, Pittsburgh, PA) according to manufacturer's recommendations. Recombinant SPARC was immobilized on a Sensor NTA chip to approximately 100 RU. Association was performed at room temperature for 5 minutes with human or bovine serum albumin (Sigma-Aldrich) at 20 ul/minute with indicated concentrations of analyte, after which complexes were dissociated for 5 minutes. Specific binding was recorded by subtracting values from the control cell without adsorbed ligand. Data were analyzed with BIAevaluation ver. 3 software by global Langmuir fitting of overlay plots for various concentrations of analyte. Binding kinetic was evaluated in at least three independent experiments and average Kd ± SD was calculated.

### Internalization and transwell assays

HUVEC cells cultured on coverslips were treated overnight in serum-free EGM-2 media with 1 ug/ml of AF488-labeled BSA and/or 1 ug/ml of SPARC labeled with AF594 using the Alexa Fluor 594 Microscale Labeling Kit (Invitrogen), rinsed with PBS and fixed with 1% paraformaldehyde at room temperature for 30 minutes. Slides were mounted in anti-fade solution, in some experiments containing Hoechst stain, and examined under a Leica DB IRB inverted fluorescent microscope (Heidelberg, Germany). To quantitatively assess internalization, HUVEC cells were cultured overnight in a 24-well plate in complete media. Cells were rinsed with PBS and treated with 1 ug/ml of AF488-labeled BSA with or without 1 ug/ml of rhSPARC in serum-free media. After indicated periods, cells were rinsed with PBS, lysates were prepared as above and intracellular proteins detected by Western blot. To study the effect of SPARC on transendothelial transport, HUVEC cells were grown for 72 hours in Transwell inserts (Corning, Corning, NY) in complete media, rinsed with PBS and 10 ug/ml of AF488-labeled BSA with or without 10 ug/ml of rhSPARC were added to the upper chamber in serum-free EGM-2 media. Transcytosed BSA was sampled from the lower chamber at various times, 12 ul of media were loaded on a 4-20% SDS-PAGE gel and BSA was detected using an anti-AF488 antibody by Western blot. For quantitative analysis, images were obtained on a Universal Hood II (Bio-Rad, Hercules, CA) and quantified with Image Lab software (Bio-Rad) by comparing densities with standards containing known amounts of proteins. All experiments were repeated at least three times and represented as an average ±SD. Statistical significance for individual time points was determined with Student's t-test.

### Lipotoxicity assay

To load BSA with palmitic acid, ultra-fatty acid free BSA (Sigma-Aldrich) was dissolved in 150 mM NaCl at 37°C to a 0.34 mM concentration and filtered. Palmitic acid (Avanti Polar Lipids, Alabaster, AL) was dispersed in 150 mM NaCl with stirring at 70°C for 30 minutes to a 2 mM concentration. Equal volumes of each solution were mixed together, stirred at 37°C for 1 hour and pH was adjusted to 7.4. Aliquots of this stock solution containing 1 mM palmitic acid / 0.17 mM BSA were stored in dark glass vials at -20°C. Identically prepared and stored FA-free 0.17 mM BSA solution was used as the control. To assess toxicity, neuroblastoma cell lines were grown overnight in 96-well plates to ~50% confluency in RPMI media supplemented with 5% FBS. Palmitic acid loaded onto BSA and BSA control were added to a final concentration of 0 - 200 uM. Cells were incubated for 24 hours under either normoxic or hypoxic conditions. For hypoxia, cells were incubated in a hypoxia chamber (COY Laboratory Products, Grass Lake, MI) at 1% oxygen. Cell apoptosis was assessed by measuring activity of caspase-3 using Caspase-3 DEVD-R110 Fluorometric HTS Assay Kit (Biotium, Hayward, CA) as recommended by the manufacturer. Readings were obtained via Gen 5 software on a Synergy 2 plate reader (BioTek, Winooski, VT). Each concentration was tested in triplicate in at least three independent experiments.

### Statistical analysis

All *in vitro* experiments were repeated at least three times and standard deviations were calculated. All animal studies had at least five mice per group and mean values of the tumor volumes, weights, and vessel densities were compared. All the quantitative values obtained in the experiments were evaluated using paired Student's t-test. A p-value of 0.05 was required to ascertain statistical significance.

## SUPPLEMENTARY MATERIALS FIGURES


