# Supplementary material for: Secreted protein acidic and rich in cysteine (SPARC) induces lipotoxicity in neuroblastoma by regulating transport of albumin complexed with fatty acids
Source: Oncotarget. 2016 Oct 20;7(47):77696–706. doi: 10.18632/oncotarget.12773 (PMC5363614; doi:10.18632/oncotarget.12773)
Supplement: Supplementary file 1 [file oncotarget-07-77696-s001.pdf]

Secreted protein acidic and rich in cysteine (SPARC) induces lipotoxicity in neuroblastoma by regulating transport of albumin complexed with fatty acids

SUPPLEMENTARY FIGURES

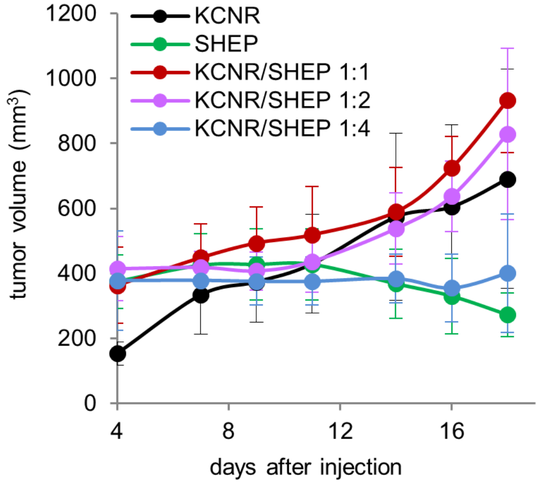

Supplementary Figure S1: *In vivo* model of stroma-rich neuroblastoma. Experimental stroma-rich tumors were established in immunodeficient mice by subcutaneous injection of a mixture of tumorigenic KCNR cells and non-tumorigenic (stromal) SHEP cells at the indicated ratios, or each type of cells alone. Tumor progression was inhibited at the 1:4 ratio, when stromal cells comprised 75% of the tumor mass with p<0.05 after day 16.

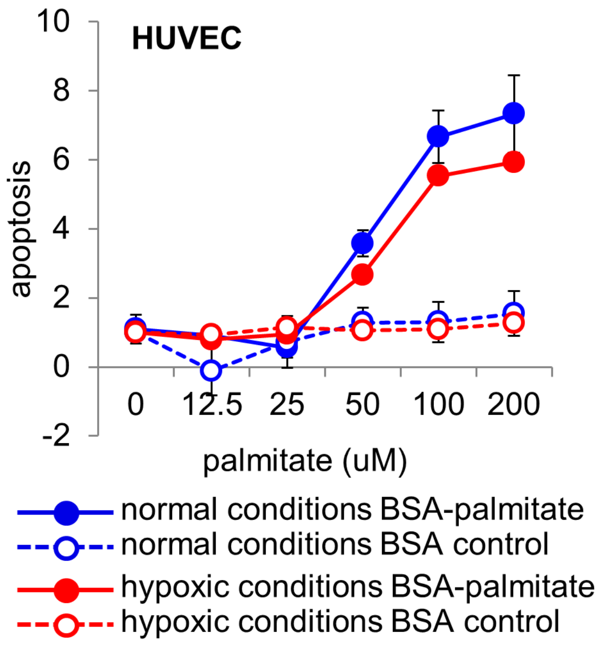

Supplementary Figure S2: Hypoxia does not increase lipotoxicity in normal cells. Lipotoxicity of saturated palmitic acid was tested in normal HUVEC cells. Palmitate caused lipotoxicity in normal cells at normal levels of oxygen, but in contrast to cancer cells, lipotoxicity was not enhanced in hypoxic conditions.
